# Complete Genome Sequence of *Methanosphaerula palustris* E1-9C^T^, a Hydrogenotrophic Methanogen Isolated from a Minerotrophic Fen Peatland

**DOI:** 10.1128/genomeA.01280-15

**Published:** 2015-11-05

**Authors:** Hinsby Cadillo-Quiroz, Patrick Browne, Nikos Kyrpides, Tanja Woyke, Lynne Goodwin, Chris Detter, Joseph B. Yavitt, Stephen H. Zinder

**Affiliations:** a School of Life Sciences, Arizona State University, Tempe, Arizona, USA; b Swette Center for Environmental Biotechnology at the Biodesign Institute, Arizona State University, Tempe, Arizona, USA; c Department of Energy, Joint Genome Institute, Walnut Creek, California, USA; d Los Alamos National Laboratory, Los Alamos, New Mexico, USA; e Department of Natural Resources, Cornell University, Ithaca, New York, USA; f Department of Microbiology, Cornell University, Ithaca, New York, USA

## Abstract

Here, we report the complete genome sequence (2.92 Mb) of *Methanosphaerula palustris* E1-9C^T^, a methanogen isolated from a minerotrophic fen. This is the first genome report of the *Methanosphaerula* genus, within the *Methanoregulaceae* family, in the *Methanomicrobiales* order. E1-9C^T^ relatives are found in a wide range of ecological and geographical settings.

## GENOME ANNOUNCEMENT

*Methanosphaerula palustris* E1-9C^T^, a hydrogenotrophic methanogen, isolated from a minerotrophic fen (1), was identified as a novel species and genus (2) in the family *Methanoregulaceae* (3). All five species of the *Methanoregulaceae* were isolated from different environments, including acidic or neutral peatlands, bioreactors, or rice fields (3). Physiological variability has been observed across the five species, and the genetic basis of these differences is not yet clearly understood. For instance, E1-9C^T^ and *Methanoregula boonei* 6A8^T^ were isolated from contrasting peatlands, and showed significant morphological and growth differences (2). Moreover, the abundance of their related phylotypes showed differential distribution in nearby peatlands: E1-9C^T^ types were dominant in minerotrophic fen and minimal in acidic oligotrophic bog, while 6A8^T^ had the opposite pattern (1). The functional and genomic basis for their differential distribution requires further evaluation. Here, we provide the first genome report in the genus *Methanosphaerula*.

The E1-9C^T^ genome was completed by Sanger sequencing (6- and 40-Kb fosmid libraries) and 454 pyrosequencing, producing a 9× coverage of the final genome. The *Phred/Phrap/Consed* software was used for sequence assembly and quality assessment (4–6). Possible misassemblies were corrected with Dupfinisher (7) or transposon bombing of bridging clones (Epicentre). Gaps between contigs were closed by *Consed* editing, primer walking, or PCR amplification. Evaluation of functional annotations and comparative analyses were done using the Integrated Microbial Genomes (IMG-ER) platform (8).

The genome sequence length was 2,922,917 bp with a G+C content of 55.35%. The genome contains 2,792 protein-coding sequences, 137 pseudogenes, 55 tRNA genes, and 3 complete rRNA operons. A total of 64.5% of the open reading frames (1,844) are protein-coding genes with function predictions.

Analysis of NCBI Clusters of Orthologous Groups (COG) categories showed the presence of all genes involved in hydrogenotrophic methanogenesis, as well as genes for formate dehydrogenase and formate transport. This supports observations of only H_2_/CO_2_ and formate utilization for growth and methane production (2). A new putative trait, also recently identified in *M. boonei* 6A8^T^ (9), is the presence of three redundant mechanisms for K^+^ transport (trk, kup, and kdp). The ATP-driven kdp K^+^ uptake system is common in bacteria and its presence in methanogens has been suggested as the product of horizontal gene transfer (9). The E1-9C^T^ genome shows the presence of 244 putative transporters, where some have no homology to equivalent transporters in close relatives, as in the case of two sets of molybdate transporters (loci: Mpal_210-212, Mpal_1582-1585) in the ABC transporters superfamily. Molybdenum is required for early enzymatic steps of some hydrogenotrophic or formate-utilizing methanogens (10), and structural differences in transporters could play roles on specificity or affinities with possible consequences in the microbe’s lifestyle.

The E1-9C^T^ genome includes one CRISPR locus, along with nine CRISPR-associated genes. This feature is absent in 6A8^T^, although it is present in the close relative *Methanolinea tarda* NOBI-1^T^. This suggests differential dynamics for genomic interactions with exogenous sources, including viruses or plasmids (11), among closely related methanogens.

Further comparative analyses of multiple *Methanoregulaceae* genomes, or species within other taxa, will provide insights into diverse traits of methanogens.

### Nucleotide sequence accession numbers.

This whole-genome shotgun project has been deposited in DDBJ/EMBL/GenBank under the accession number CP001338. The version described in this paper is the first version, CP001338.1.
